# Investigation of the Microstructural and Mechanical Properties of Fiber-Reinforced Roller-Compacted Concrete Under High-Temperature Exposure

**DOI:** 10.3390/ma18112430

**Published:** 2025-05-22

**Authors:** Murteda Ünverdi, Yahya Kaya, Naz Mardani, Ali Mardani

**Affiliations:** 1 Department of Civil Engineering, Faculty of Engineering, Bursa Uludag University, 16059 Bursa, Turkey; murtedaunverdi@uludag.edu.tr (M.Ü.); 512126007@ogr.uludag.edu.tr (Y.K.); 2 Department of Mathematics Education, Faculty of Education, Bursa Uludag University, 16059 Bursa, Turkey; nazmardani@uludag.edu.tr

**Keywords:** roller-compacted concrete, high-temperature performance, microstructure, compressive strength, fiber reinforcement

## Abstract

In this study, the effects of different fiber types on improving the high-temperature performance of roller-compacted concrete (RCC) were comprehensively investigated. For this purpose, 60 mm long steel (S), polypropylene (PP), and environmentally sustainable waste steel (WS) fibers were incorporated into RCC at volumetric ratios of 0%, 0.25%, 0.50%, 0.75%, 1.00%, and 1.25%. The prepared specimens were exposed to controlled conditions at 25 °C (room temperature), 300 °C, 600 °C, and 900 °C, and the influence of thermal exposure on compressive strength and permeability characteristics was thoroughly evaluated. The findings revealed that high temperatures led to significant changes in the physical and mechanical properties of the concrete. Notably, at elevated temperatures such as 600 °C and 900 °C, S and WS fibers were found to reduce strength loss by limiting the propagation of microcracks within the concrete matrix. However, PP fibers were observed to lose their effectiveness at high temperatures due to melting in the range of approximately 160–170 °C, which negatively affected mechanical performance. One of this study’s key findings is that waste steel fibers offer a sustainable alternative while exhibiting comparable performance to conventional steel fibers. These results highlight the potential of recycling industrial waste to reduce environmental impact and lower overall costs.

## 1. Introduction

The increasing infrastructure investments and fire safety requirements in the construction sector necessitate a detailed investigation of the performance of concrete under high temperatures [1,2,3]. In particular, the thermal behavior of roller-compacted concrete (RCC) used in critical applications such as power plants, industrial facilities, and transportation structures, where high-temperature exposure is likely, stands out as a key factor in structural safety [4,5]. In this context, RCC has become increasingly preferred due to its technical and economic advantages compared to conventional vibrated concrete [6]. Studies in the literature have shown that RCC can provide 30–50% higher compressive strength [7,8], 15–40% lower cost [9,10], and shorter construction durations [11,12,13]. However, the characteristically dense structure of RCC, while contributing to high compressive strength, also results in notable disadvantages such as low tensile strength [14] and limited toughness [15]. These drawbacks have led researchers to explore the use of fiber reinforcement to improve the mechanical performance of RCC [16,17]. Fiber reinforcement enhances the tensile strength of concrete, limits crack formation, and improves thermal stability under high-temperature conditions.

Fiber reinforcement is essential for enhancing concrete’s ductility and energy absorption capacity [16,17]. Incorporating fibers into the concrete matrix delays crack initiation and controls crack propagation, significantly improving impact resistance [13,18]. Today, various types of fibers such as steel (S), polypropylene (PP), glass, plastic, and carbon are commonly used in fiber-reinforced concrete applications [19,20,21,22,23]. These fibers significantly enhance mechanical properties such as impact resistance, tensile strength, abrasion resistance, and fire resistance [24,25,26,27].

Advancements in fiber-reinforced concrete technology not only improve material efficiency and reduce waste but also support sustainable construction practices [28]. However, using steel fibers in RCC presents a major sustainability drawback due to the high CO_2_ emissions generated during production [20,29]. As a solution to this problem, alternative fibers produced from natural or recycled materials are becoming increasingly common. The use of waste steel (WS) fibers in cementitious systems provides significant contributions to sustainable infrastructure development efforts. While the production of traditionally used industrial steel fibers leads to high energy consumption and carbon emissions, the evaluation of waste-sourced fibers has the potential to reduce this environmental burden [6]. In particular, the use of steel fibers obtained from tire recycling as concrete reinforcement allows both the reduction of waste and the protection of natural resources. This approach stands out as a material management strategy compatible with circular economy principles in line with environmental sustainability purposes [20]. In addition, life cycle assessments (LCAs) show that greenhouse gas emissions and total energy consumption are significantly reduced during the production process of concretes containing waste steel fibers [6]. In this context, the use of waste steel fibers in infrastructure projects constitutes a sustainable alternative in terms of both technical performance and environmental impact. Research in the literature has shown that WS fibers yield favorable results regarding strength and durability and provide significant potential when evaluated within the framework of circular economy principles [30,31]. These findings are essential for the development and widespread adoption of sustainable concrete technologies.

A literature review reveals that studies comprehensively addressing the high-temperature performance of RCC are quite limited. Among the existing studies, the work by Hejazi et al. [32] stands out as the only comprehensive investigation that systematically examined the thermal behavior of RCC within the 40 °C to 80 °C range. In that study, it was found that in RCC reinforced with S and PP fibers, both fiber types significantly improved thermal stress resistance, flexural strength, and thermal crack resistance. Notably, the high coefficient of thermal expansion of PP fibers (≈100 × 10^−6^/°C) was reported to delay thermal cracking by increasing tensile stresses in the concrete matrix. However, the study’s relatively low temperature range (maximum 80 °C) and the exclusion of extreme conditions such as fire (300–900 °C) limit the full understanding of RCC’s behavior under high-temperature exposure. Understanding how RCC behaves under elevated temperatures is critical for developing durable and safe construction materials capable of withstanding extreme thermal loads such as fire [5,33,34]. Therefore, more comprehensive research is needed to reveal RCC’s mechanical and microstructural changes under high-temperature conditions.

In evaluating the high-temperature performance of fiber-reinforced RCC (FR-RCC), it is essential to examine mechanical properties and microstructural changes thoroughly. The literature has shown that high temperatures can significantly alter the micro- and macrostructure of concrete composites, reducing mechanical performance [35,36]. The effects of different fiber types on pore structure, crack propagation mechanisms, and phase transitions can be analyzed in detail using advanced imaging techniques. Such microstructural analyses provide crucial insights into the effects of fiber reinforcement on the spalling resistance of concrete. Moreover, these analyses can contribute to developing material design strategies to enhance the strength and long-term performance of FR-RCC under extreme heat conditions such as fire [37,38]. Studies have shown that fiber types exhibit different performances in terms of fire resistance. In particular, the fire behavior of fibers with low melting temperatures differs from that of fibers with high melting temperatures such as steel [37,38].

Critical microstructural changes occur within specific temperature ranges when concrete is exposed to high temperatures. These include the loss of chemically bound water between 100 and 300 °C, the decomposition of calcium hydroxide (Ca(OH)_2_) between 400 and 600 °C, and the breakdown of calcium silicate hydrate (C-S-H) gel above 600 °C, all of which play a key role in determining the mechanical and microstructural properties of concrete [39]. These thermal degradation processes cause thermal incompatibilities at the aggregate–matrix interface and, when combined with changes in pore structure, negatively affect the material’s overall performance [40,41]. These comprehensive microstructural analyses form a critical foundation for determining the optimum fiber type and dosage for fire-resistant concrete design, enhancing thermal shock resistance, and developing long-term durability prediction models [34].

In this study, a fiber-reinforced composite material (FR-RCC) was developed to improve RCC’s mechanical properties and thermal resistance, and its high-temperature performance was comprehensively investigated. In this context, the thermal stability, structural integrity, and mechanical performance of RCC incorporating steel (S), polypropylene (PP), and waste steel (WS) fibers were systematically evaluated within a temperature range of 25–900 °C. The original contribution of this study lies in the comparative evaluation of WS fibers obtained from industrial waste, offering a practical alternative from economic and environmental sustainability perspectives. In the experimental program, fibers with a length of 60 mm were used at five different volumetric ratios ranging from 0% to 1.25%. The produced specimens were thoroughly analyzed regarding strength and permeability performance after exposure to high temperatures. The results quantitatively revealed the effects of different fiber types on high-temperature performance and aimed to fill essential gaps in the literature regarding the thermal behavior of FR-RCC. This comprehensive evaluation provides critical data for fire-resistant infrastructure design and contributes to developing sustainable construction materials.

## 2. Materials and Methods

### 2.1. Materials

In this study, CEM I 42.5 R-type cement conforming to the EN 197-1 standard [42] was used as the binder material. The chemical composition, physical, and mechanical properties of the cement were provided by the manufacturer, and the relevant data are summarized in Table 1.

**Figure 1:**
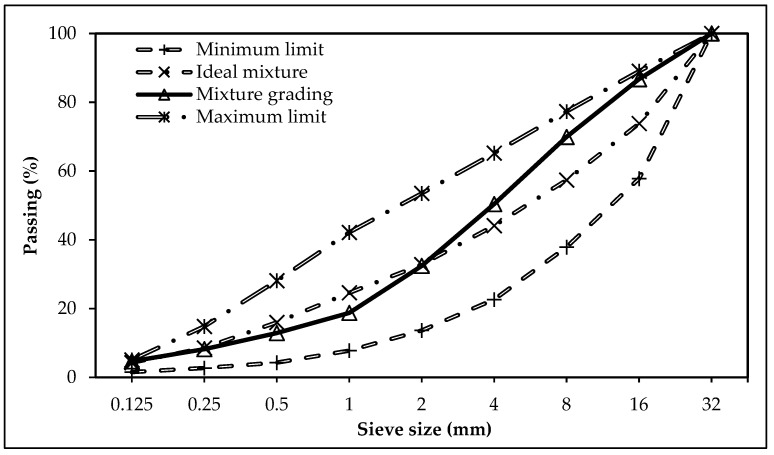
Aggregate gradation curve according to TS 802 standard [45].

In this study, crushed limestone aggregates with particle sizes of 0–5 mm, 5–12 mm, and 12–22 mm were used. The water absorption capacity, saturated surface-dry (SSD) unit weight, and loose bulk density of the aggregates were determined by EN 1097-6 [43], and the relevant data are presented in Table 2. The aggregate gradation was determined based on the sieve analysis results conducted according to EN 933-1 [44]. Accordingly, the optimized aggregate blend used to produce RCC consisted of 60% 0–5 mm, 20% 5–15 mm, and 20% 15–25 mm aggregates by weight. The prepared aggregate concrete’s particle size distribution was within the gradation limits specified in TS 802 [45], as illustrated in Figure 1.

**Figure 2:**
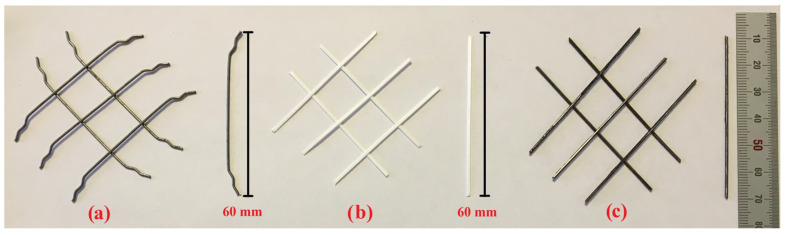
Fibers used in the RCC: (**a**) 60 mm steel; (**b**) 60 mm PP; (**c**) 60 mm WS fiber.

In RCC, hooked-end steel, polypropylene (PP) and waste steel (WS) fibers, each 60 mm long, were used (Figure 2). Waste steel fibers were obtained by separating tire waste from plastics. Accordingly, they were used by being subjected to a temperature of approximately 100 degrees and then purified from plastics. Specific physical and mechanical properties of S, PP and WS fibers are presented in Table 3 based on the data provided by the manufacturers. The fibers were added to the concrete at different volumetric ratios of 0%, 0.25%, 0.50%, 0.75%, 1%, and 1.25%.

### 2.2. Methods

#### 2.2.1. Preparation of Concrete

The RCCs were prepared in a four-stage process according to the ASTM C1435 standard [46]: (1) mixing the aggregates for 30 s, (2) an additional 30 s of mixing with the cement, (3) adding fibers in the specified proportions and mixing for an additional 30 s, and (4) final mixing for 5 min after adding water [46].

The prepared concrete mixes were cast in two layers for 150 mm cubic samples and three layers for 150 × 300 mm cylindrical samples. Each layer was compacted for 20 s using a vibrating hammer [47]. After being left in the molds for 24 h, the samples were water cured at a temperature of 23 ± 2 °C. Three replicate samples were produced for each concrete, with the control mixture referred to as “C” and the fiber-reinforced concrete named with standard codes such as “S60-0.50” (a concrete containing 60 mm long and 0.50% steel fibers).

#### 2.2.2. Optimum Water Content and Mixture Proportion

**Figure 3:**
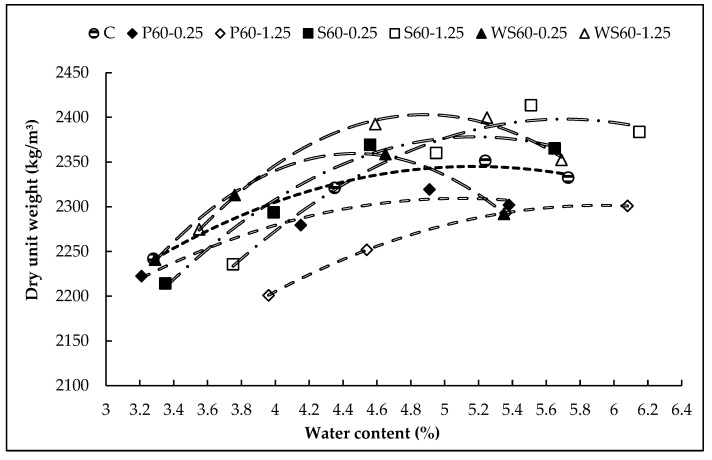
The relationship between the optimum water content and maximum dry unit weight of the concrete mixtures.

The RCCs were designed using the maximum density method following the ACI 207.5R.99 standard [48], while keeping the cement content fixed at 300 kg/m^3^. Four different mixtures with a water-to-cement (w/c) ratio between 0.37 and 0.52 were prepared to determine the optimum water content. To determine the dry unit volume weight of the RCC, 600 g samples taken from the compacted specimens were dried at 105 °C until they reached a constant weight [7,47], and the water content (*w*) was calculated using Equation (1).

(1) $$w=\frac{m_{wet}-m_{dry}}{m_{dry}}\;\times \;100$$

where w is water content, *m_wet_* is the wet mass of the concrete, and *m_dry_* is the dry mass of the concrete. The fresh unit weight (*γ_wet_*) was determined by the ratio of sample mass (*m*) to volume (*v*), and the dry unit weight (*γ_dry_*) was calculated using Equation (2):

(2) $$\gamma_{dry}=\frac{\frac{m}{v}}{1+w}$$

where *γ_dry_* is the dry unit weight, *m/v* is *γ_wet_*, and w is the water content. Based on the calculated parameters, curves showing the relationship between optimum water content and maximum dry unit weight were created, and the optimum water content corresponding to the maximum density was determined from these curves [47]. For example, the curves for fiber-reinforced concrete at 0.25% and 1.25% fiber content are shown in Figure 3. The required theoretical and corrected material quantities for 1 m^3^ of RCC are summarized in Table 4 and Table 5. This method was applied to achieve maximum density while targeting the minimum void ratio in the mixture design [48].

#### 2.2.3. Experimental Study

##### Consistency

The workability of RCC mixtures was evaluated using the Vebe test according to Procedure A of ASTM C1170 standard [49]. During the test, the time taken for the mortar ring around the mold to fill completely was measured by applying a 22.7 kg pressure plate, and this time was used as the key parameter for determining the mix consistency.

##### Properties of Hardened Concretes

**Figure 4:**
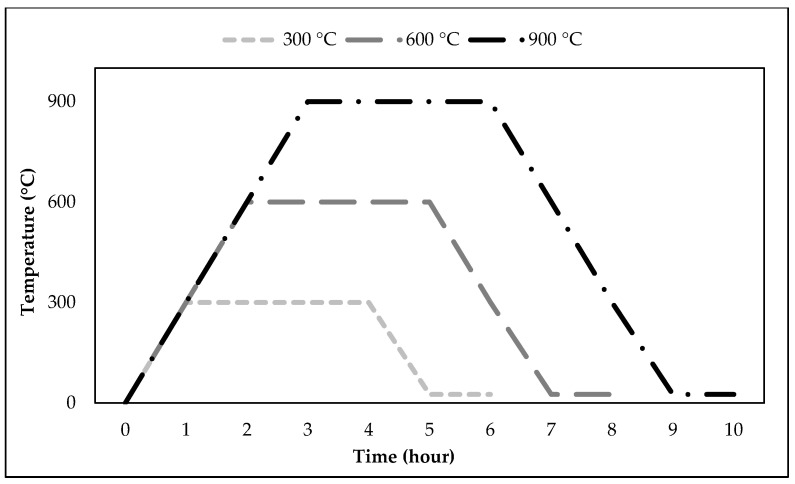
High-temperature resistance test process [52].

The high-temperature resistance of RCC was investigated through damage analysis, weight loss, compressive strength, and water absorption capacity changes on samples exposed to different temperature levels. The effect of high temperature was studied by exposing the samples to controlled temperatures of 300 °C, 600 °C, and 900 °C after they had been dried at 105 °C for 24 h following a 28-day water curing period. The 28-day curing period for concrete is a standard benchmark for evaluating strength because concrete achieves approximately 95–99% of its full strength within this time. The thermal treatment process was conducted at a heating rate of 5 °C per minute, and once the target temperature was reached, the samples were maintained at a constant temperature for 180 min (Figure 4).

The 28-day compressive strength and water absorption capacity of the concretes were determined on 150 mm cube samples, in accordance with EN 12390-3 [50] and ASTM C642 standards [51], respectively.

#### 2.2.4. Statistical Analysis

In this study, the Taguchi method and ANOVA analysis were applied to optimize RCC’s strength and durability performance. In the Taguchi approach, three different signal-to-noise (*S*/*N*) ratio calculation methods are based on the performance characteristic: nominal is best, larger is better, and smaller is better [53]. In this study, the “larger is better” criterion provided in Equation (3), proposed by Mandal et al. [54], was used for parameters requiring maximization, such as compressive strength.

(3) $$\;\frac{S}{N}=\;-10log(\frac{1}{n}\;\sum_{i=1}^{n}{1/yi}^{2})$$

Here, *yi* represents the observed data in the *i*-*th* experiment, and *n* refers to the number of observations, *i* refers to observation numbers (1, 2, 3, etc.), and *S*/*N* is the signal-to-noise ratio of the Taguchi approach.

The L18(6^1^3^2^) orthogonal array was chosen to examine the interactions of the factors. This experimental design provides an appropriate number of experiments for a 6-level factor (fiber dosage) and two 3-level factors (temperature and fiber type). This approach allows maximum information to be obtained with the minimum number of experiments, ensuring efficient use of resources during the experimental process. The levels of the selected factors are presented in Table 6.

## 3. Results and Discussion

### 3.1. Properties of Concrete Mixtures

#### Consistency

Based on the data presented in Table 7, an examination of the Vebe times of fiber-reinforced RCC reveals that adding fibers adversely affects the workability of the concrete, regardless of fiber type and dosage. Furthermore, it was determined that this adverse effect becomes more pronounced with increasing fiber content. This phenomenon is attributed to the fibers increasing internal friction within the mixture, thereby restricting its mobility. The observed increase in Vebe times indicates that fiber-reinforced RCC requires higher energy for placement and compaction because the presence of fibers reduces its workability and increases the friction between the concrete mixture components. This leads to denser, less fluid mixtures that demand more energy to ensure proper compaction and distribution of fibers. Similar findings have also been highlighted in the studies conducted by Ghahari et al. [55] and Haghnejad and Modarres [56].

Using 60 mm long steel fibers resulted in an increase in Vebe time ranging from 24% to 230%. This is believed to be related to the reduction in concrete mix flowability caused by the hooked-end geometry of the steel fibers (Figure 2). The negative impact of steel fibers on the workability of concrete mixtures has also been emphasized by many researchers [57,58,59].

In the case of WS fiber usage, the Vebe time was found to increase by 18% to 212%. It appears that the effect of waste steel fibers on workability is more limited than that of conventional steel fibers. This difference is thought to stem from the fact that the hooked ends of steel fibers reduce the flow performance of the concrete mix to a greater extent.

For concrete mixtures containing PP fibers, the Vebe time increased by 12% to 185%. Due to their ability to disperse more uniformly compared to S and WS fibers, PP fibers demonstrated better performance regarding Vebe time [60,61]. Nevertheless, as the content of steel and waste steel fibers increased, the interlocking of fibers became more intense, which in turn had a more detrimental effect on workability. This finding highlights the need for careful fiber content optimization to balance mechanical performance and workability. The results are consistent with previous studies conducted by Yildizel et al. [62] and Benyarar et al. [63]. When the Vebe times of fiber-reinforced concrete mix were compared with the control mixture, the most significant increase was observed in concrete mixtures containing steel fibers, followed by those with WS and PP fibers, respectively.

### 3.2. Properties of Hardened Concrete

The hardened-state properties of fiber-reinforced and unreinforced RCC exposed to different temperature levels were examined in detail in terms of surface damage observed in the specimens after high-temperature exposure, failure behavior under compressive load, changes in compressive strength, and water absorption capacity.

#### 3.2.1. Surface Damage After High-Temperature Exposure

**Figure 5:**
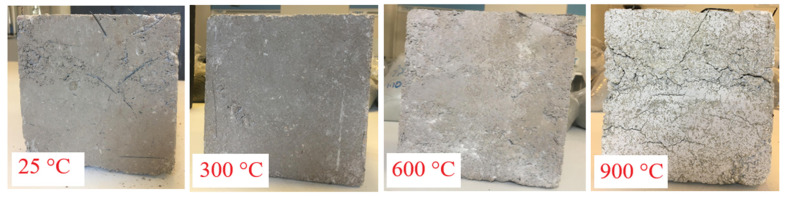
Color change of RCC specimens after high-temperature exposure.

In all RCC specimens exposed to elevated temperatures, characteristic color changes were observed in parallel with increasing temperature. These changes exhibited similar color transitions at the same temperature levels regardless of fiber type, indicating a consistent thermal response. Since the color changes were identical regardless of fiber type, only the color transition of concretes containing WS fibers is presented as a representative example. In WS fiber-reinforced specimens, a standard gray tone was observed at 25 °C, a pale yellowish hue at 300 °C, a distinct reddish color at 600 °C, and greenish-gray tones became dominant at 900 °C (Figure 5). This color evolution is directly associated with thermochemical transformations within the concrete components. The yellow tones appearing from 300 °C onward indicate the near-complete loss of physically unbound water and the onset of rapid decomposition of cement hydration products [64]. The reddish hue at 600 °C is attributed to the formation of iron oxides [36,65], while the greenish tones at 900 °C are considered to reflect mineralogical transformations in the aggregates [66]. The consistent color changes observed across all specimens suggest that thermal degradation mechanisms proceed similarly regardless of fiber type. However, with the use and increasing dosage of fibers, a more porous surface structure was observed on the specimens.

**Figure 6:**
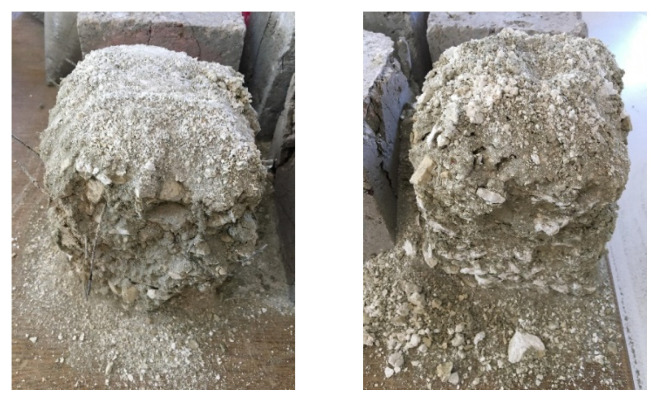
RCC specimens after exposure to 900 °C and keeping the produced sample at room conditions for 24 h.

After being exposed to 900 °C and left in ambient air for 24 h, the specimens showed noticeable structural deterioration (Figure 6). This phenomenon is primarily due to the decomposition of calcium hydroxide (Ca(OH)_2_) into calcium oxide (CaO) and water (H_2_O) at high temperatures [67]. During this chemical transformation, approximately 40% volume loss occurs. Subsequently, the CaO comes into contact with atmospheric moisture and carbon dioxide, reverting to Ca(OH)_2_ and calcium carbonate (CaCO_3_), leading to expansion [68]. These opposing reactions generate significant internal stresses within the concrete, ultimately compromising its structural integrity.

**Figure 7:**
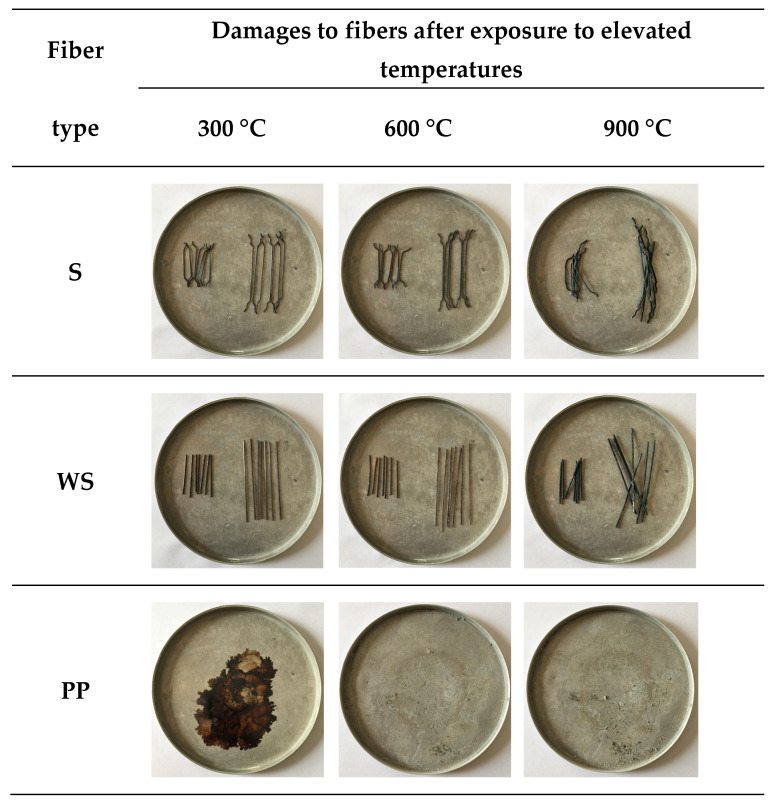
Visual changes of fibers exposed to 300, 600, and 900 °C in the RCC.

**Figure 8:**
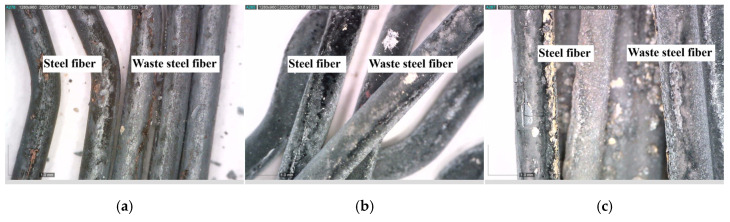
Microscopic images of the fibers used in the RCC after exposure: (**a**) 300; (**b**) 600; (**c**) 900 °C.

Thanks to the crack-bridging effect of fibers, crack propagation is largely mitigated [69]. However, this beneficial effect is directly related to the thermal resistance of the fibers [1]. Microscopic examinations conducted on fibers exposed to 300, 600, and 900 °C (Figure 7 and Figure 8) clearly reveal the structural transformations of the fibers.

While no significant deterioration was observed in S and WS fibers at 300 °C, surface cracking and local melting began to be observed in both fiber types at 600 °C. The volume expansions observed in steel fibers with the increase in temperature negatively affect the ITZ region. In addition, cracks formed on the fiber surface also weaken the ITZ. Accordingly, the ITZ behavior of steel fibers is negatively affected at temperatures of 600 and above (Figure 7 and Figure 8). Severe cracking, fragmentation, and brittle fracture behavior became evident in the fibers at 900 °C. It was determined that the fibers lost their load-carrying capacity to a great extent and their bonds with the cement matrix weakened significantly, especially at this high temperature. As for the PP fibers, they completely melted at approximately 170 °C, indicating that they showed relatively limited performance at high temperatures compared to other fiber types.

#### 3.2.2. Failure Behavior of RCC Under Compression After Thermal Exposure

**Figure 9:**
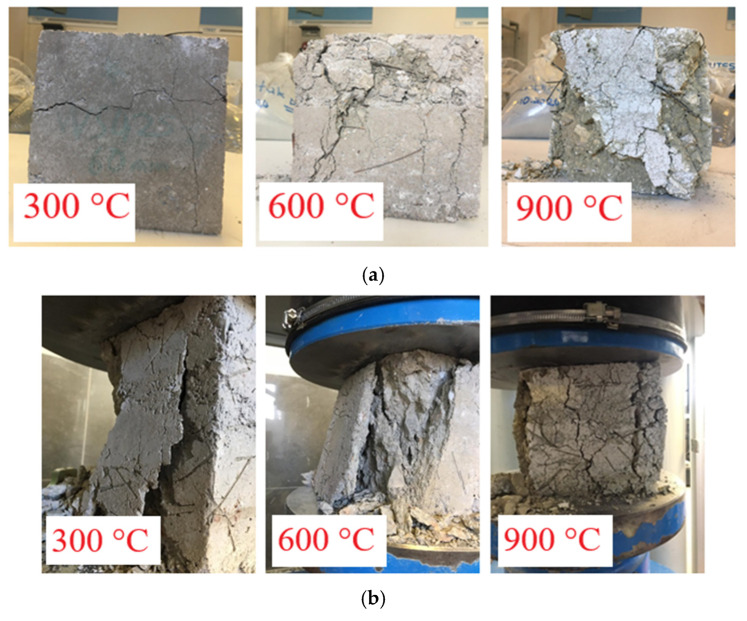
The fracture patterns of RCC specimens after the compressive strength test, following exposure to 300, 600, and 900 °C temperatures: (**a**) WS fiber-reinforced concrete; (**b**) PP fiber-reinforced concrete.

To examine the effect of fiber usage and temperature variation on the failure behavior of specimens under compressive load, the fracture patterns of the specimens were analyzed following the compressive strength test. In this context, control and fiber-reinforced RCC specimens were exposed to 300, 600, and 900 °C temperatures and subjected to compressive strength testing. The resulting fracture patterns are presented in Figure 9. Since similar fracture patterns were observed in both S and WS fiber concretes, only the fracture behavior of WS fiber-reinforced concretes is evaluated as an example.

Upon examining the fracture behavior, a clear evolution in fracture patterns was observed with increasing temperature. It was noted that the increase in tensile strength with fiber usage helped limit lateral deformations and maintained the integrity of the specimens after exposure to high temperatures. At temperatures up to 300 °C, the limited decomposition of cement hydration products [20] and the crack-bridging effect of fibers [64] helped maintain the specimen’s integrity and prevented complete disintegration. This stabilization effect was particularly noticeable in specimens reinforced with steel and waste steel fibers.

**Figure 10:**
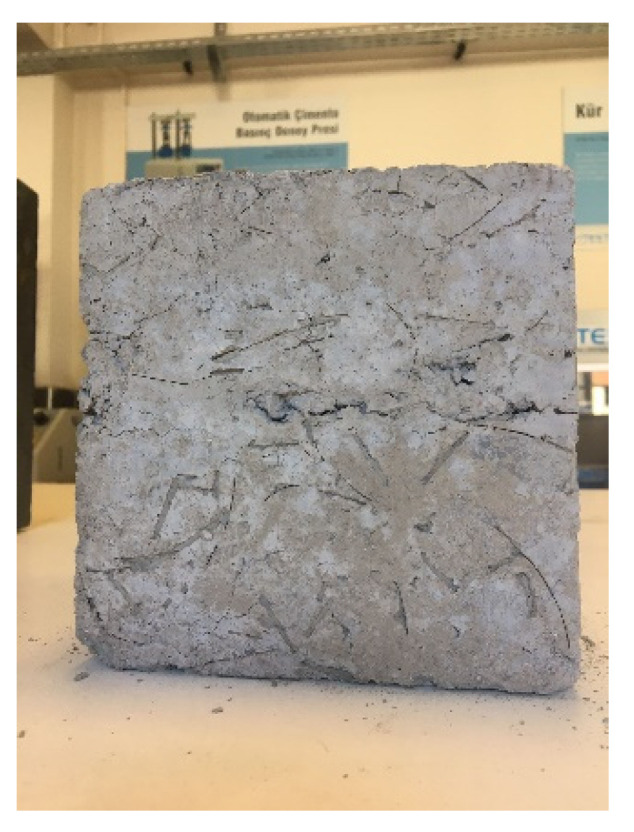
The formation of voids due to fiber melting in the PP fiber-reinforced concrete.

The behavior observed in PP fiber-reinforced concretes differed distinctly from that of other fiber types. PP fibers’ relatively low melting temperature (170 °C, Table 3) leads to the formation of characteristic micro-void networks within the concrete matrix (Figure 10). These voids act as stress concentration regions during compression testing, facilitating crack formation and significantly disrupting the surface integrity, especially in mixtures with high dosages such as 1.25% (Figure 9). However, paradoxically, this melting behavior can also offer an advantage for high-temperature performance. Literature data indicate that the void networks formed by the melting of PP fibers facilitate the dispersion of vapor pressure that accumulates in concrete at temperatures above 300 °C, thus enhancing spalling resistance. This dual effect of PP fibers deteriorating mechanical performance at room temperature while improving spalling resistance at high temperatures requires careful optimization in engineering applications.

In the 400–600 °C range, the dehydroxylation of Ca(OH)_2_ [15] initiated structural degradation, and above 600 °C, microstructural damage caused by the decomposition of limestone aggregates [70] significantly increased crack formation. A significant decrease in the crack prevention capacity of S and WS fibers was observed at this temperature level. The mismatch between the thermal expansion coefficients of the fibers and the concrete matrix led to microcracks at the interface, which adversely affected the dimensional stability of the specimens [71,72]. As the temperature increased, specimens were observed to crush and disintegrate, showing that fiber reinforcement plays a critical role in concrete performance under high-temperature conditions.

In PP fiber-reinforced concretes, the situation is more critical. The voids formed due to the complete melting of PP fibers at 170 °C severely disrupt the material integrity at 600 °C (Figure 9). The dense crack networks and structural disintegration observed in these concretes show that PP fibers become ineffective at this temperature level.

Specimens exposed to 900 °C exhibited severe structural deterioration. Compressive strength testing revealed that the specimens almost completely disintegrated, and cracks spread extensively. The primary cause of this was the evaporation of chemically bound water in the concrete matrix, leading to a critical increase in porosity [73]. Furthermore, the substantial degradation of C-S-H gel and the decomposition of calcium carbonate (CaCO_3_) caused irreversible damage to the material’s internal structure [74,75]. However, these adverse effects were partially mitigated in S and WS fiber-reinforced specimens. In PP fiber concretes, the concrete’s external shell and internal structure were nearly completely shattered (Figure 9).

In this study, the performance of RCC under high-temperature conditions was evaluated based on changes in compressive strength and water absorption capacity. The results of compressive strength and water absorption capacity for RCC exposed to different temperatures are summarized in Table 8. Experimental results show significant increases in water absorption capacity with rising temperatures. At 300 °C, the observed increase in water absorption ranged from 40 to 57%, and by the time the temperature reached 900 °C, the increase had risen to between 177 and 287%. Losses in compressive strength ranged from 25 to 35% at 600 °C, and at 900 °C, this loss increased to 84–93%. Notably, significant weight losses were observed in mixtures containing PP fibers, ranging from 9 to 16% at 900 °C.

#### 3.2.3. The Change in Compressive Strength of RCC After Exposure to High Temperatures

To examine the effect of fiber usage and temperature variation on the failure behavior of specimens under compressive load, the fracture patterns of the specimens were analyzed following the compressive strength test. In this context, control and fiber-reinforced RCC specimens were exposed to 300, 600, and 900 °C temperatures and subjected to compressive strength testing. The resulting fracture patterns are presented in Figure 9. Since similar fracture patterns were observed in both S and WS fiber concretes, only the fracture behavior of WS fiber-reinforced concretes is evaluated as an example.

**Figure 11:**
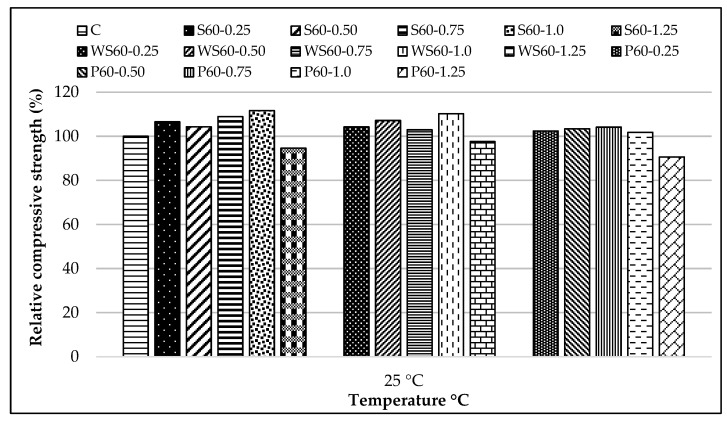
The relative compressive strength results of all RCC at 25 °C, compared to the control mixture.

The relative compressive strength results of all RCCs at 25 °C, compared to the control mixture, are shown in Figure 11.

**Figure 12:**
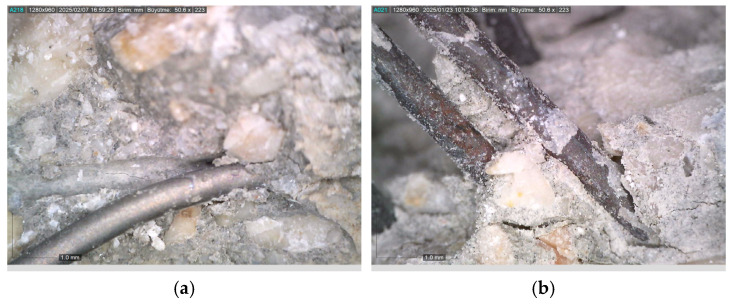
Clustering and crack formations due to excessive fiber content: (**a**) steel fiber-reinforced concrete; (**b**) WS fiber-reinforced concrete.

When the samples at 25 °C were examined, a general increase in compressive strength was observed for fiber-reinforced RCC, regardless of the fiber type. This increase was attributed to reduced horizontal stresses and the prevention of crack propagation, thanks to the fibers [19,76]. S fibers led to a maximum strength increase of 11%, WS fibers to 9%, and PP fibers to 4%. Other researchers have also reported similar results [77,78]. In terms of compressive strength, the optimum fiber content for S and WS fiber-reinforced RCC was determined to be 1.0%, while for PP fiber-reinforced concretes, it was 0.75%. It was observed that exceeding these proportions led to a decrease in compressive strength. Other researchers reported similar results [79,80]. This phenomenon is believed to be due to the difficulty in achieving a homogeneous fiber distribution as the fiber content increases [20], reduced workability [30], and the formation of fiber clusters [81]. Fiber clustering at a ratio of 1.25% in S and WS fiber-reinforced concretes can be seen in Figure 12.

**Figure 13:**
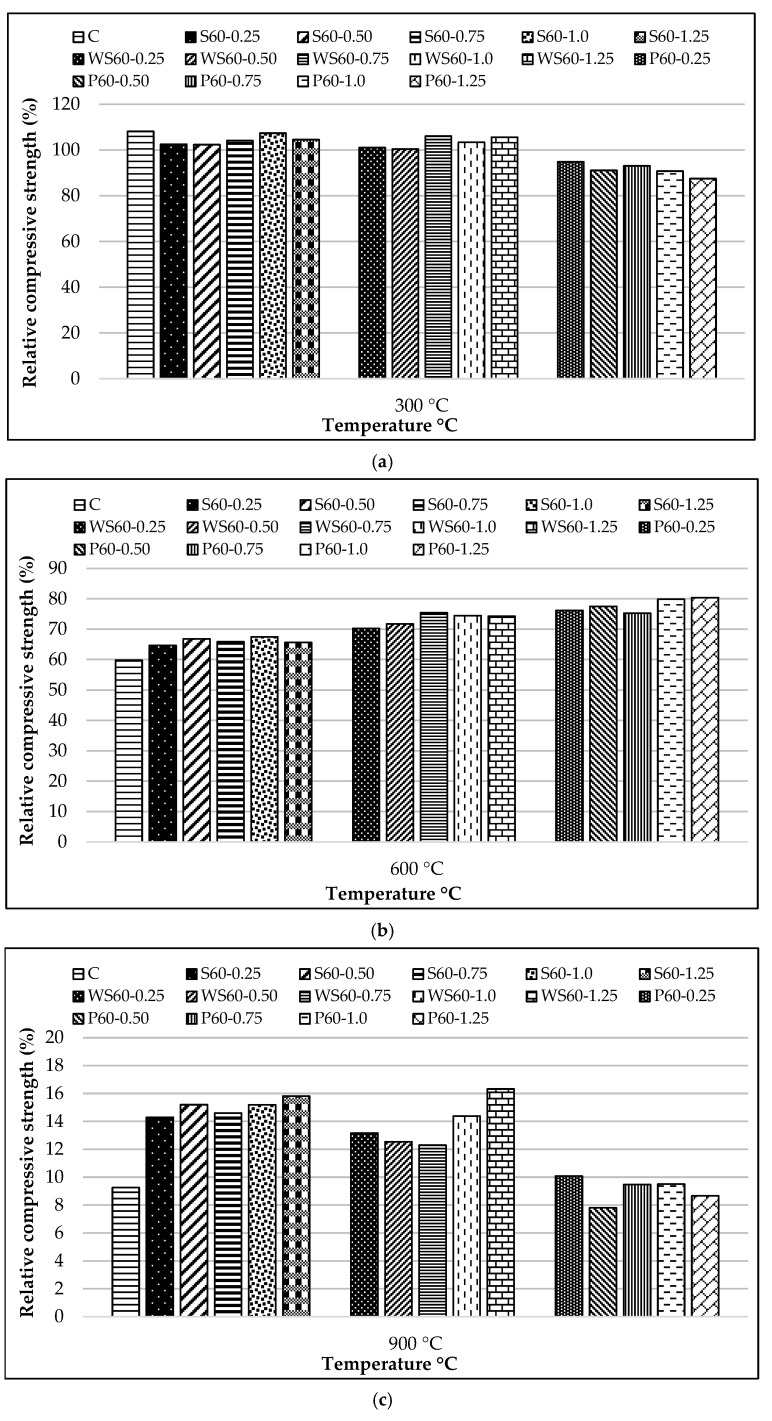
Relative compressive strength of specimens exposed to different temperatures compared to those at 25 °C: (**a**) 300; (**b**) 600; (**c**) 900 °C.

The relative compressive strength results of the specimens exposed to 300, 600, and 900 °C temperatures, compared to those at 25 °C, are presented in Figure 13a–c.

When the compressive strength of fiber-reinforced RCC exposed to 300 °C was examined, an 8% higher strength increase was recorded compared to the control mixture. This can be explained by the strengthening of the Van der Waals forces within the matrix due to the temperature rise [82], the progress of hydration of non-hydrated cement grains, and the formation of tobermorite gel with a more compact structure compared to the C-S-H phase [83].

**Figure 14:**
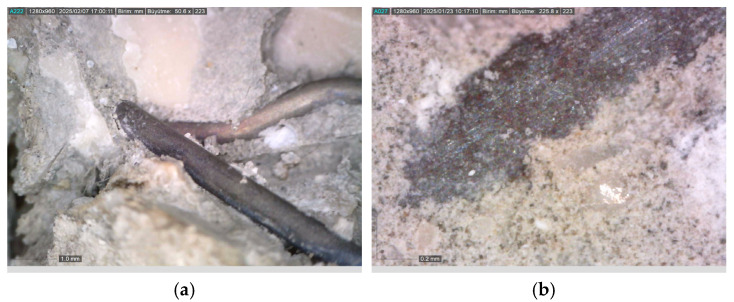
Formation of a compact structure between the fibers and the matrix: (**a**) steel fiber; (**b**) waste steel fiber.

Ji et al. [83], Shaikh and Taweel [84], Liao et al. [85], and Sellevold and Bjontegaard [86] reported that the high-temperature resistance of fiber-reinforced concrete mixtures is highly dependent on the melting point, surface properties, and thermal behavior of the fibers. In line with the aforementioned studies, a 2–7% and 1–6% strength increase was observed in S and WS fiber samples, respectively (Figure 13a). This increase is believed to be related to the densification of the C-S-H phase and the strengthening of fiber–matrix adhesion due to the previously mentioned factors [83]. As shown in Figure 14, the compact structure of the fiber–matrix interface in S and WS fiber-reinforced concretes exposed to 300 °C supports this hypothesis. Furthermore, the coefficient of thermal expansion of cement paste (10 × 10^−6^/°C) being close to that of steel fibers (8.2 × 10^−6^/°C) helps maintain the fiber–matrix adhesion under temperature effects [86,87]. Even though it is close, the expansion of steel fibers is different from concrete and can weaken the ITZ area by expanding under high temperatures.

**Figure 15:**
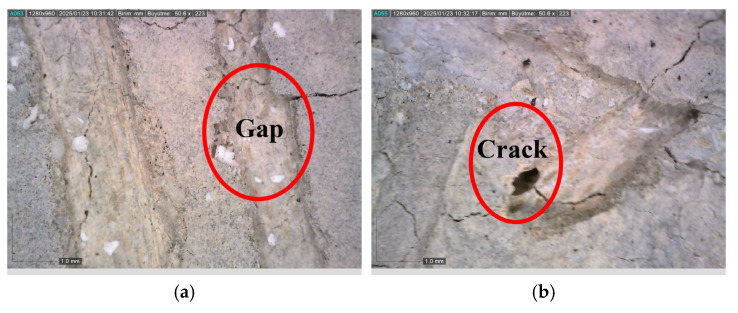
PP fiber-reinforced concretes exposed to 300 °C: (**a**) void caused by fiber melting; (**b**) crack caused by osmotic pressure.

The increase in temperature up to 300 °C negatively affected the compressive strength of PP fiber-containing RCC, which became more pronounced as the fiber dosage increased. In PP fiber concretes, a decrease in compressive strength of 6–14% was observed as the temperature rose from 25 °C to 300 °C. In the literature, it has been reported that the melting of PP fibers at 160–170 °C creates voids that provide space for osmotic pressure, thus increasing high-temperature resistance [36]. However, the opposite was observed in this study, where strength loss is believed to be caused by micro-voids created by the melting of fibers and secondary cracks caused by vapor pressure (Figure 15).

**Figure 16:**
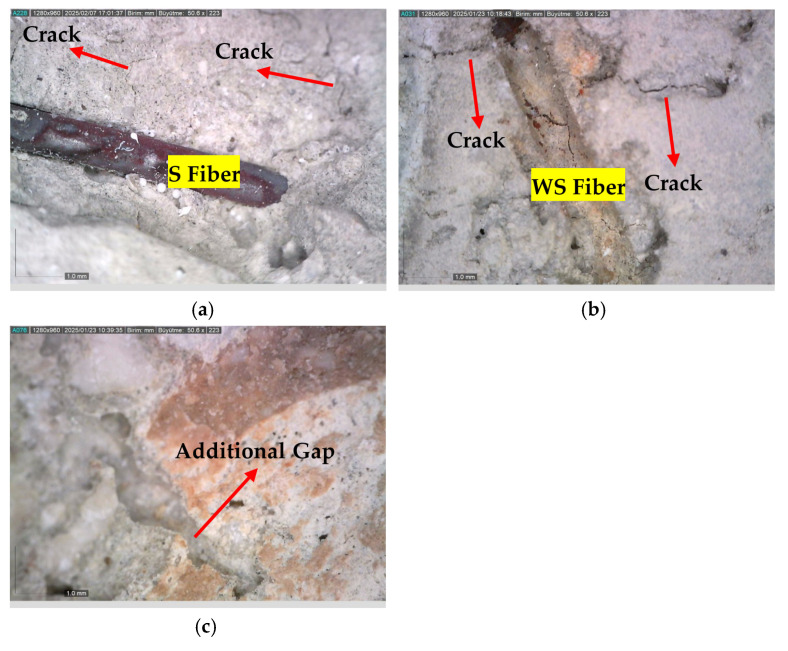
Appearance of fibers in the matrix in concretes exposed to 600 °C: (**a**) S fiber-reinforced concrete; (**b**) WS fiber-reinforced concrete; (**c**) PP fiber-reinforced concrete.

As the temperature rose from 25 °C to 600 °C, 40%, 33%, 27%, and 22% compressive strength reductions were detected for the control, S, WS, and PP fiber concretes, respectively (Figure 13b). Regardless of the fiber type, it was found that the strength loss due to high temperatures in fiber-containing mixtures was lower than that of the control mixture. It is known that the chemically bound water in calcium hydroxide (Ca(OH)_2_), formed as a result of hydration in concrete mixtures, turns into water vapor when the temperature reaches 500 °C, causing calcium oxide (CaO) to form [88]. The transformation of Ca(OH)_2_ into quicklime and water vapor does not cause a significant strength loss. Still, the evaporation of water bound to lime creates internal stresses, and the expansion resulting from the slaking of lime during cooling causes damage [89,90]. Since the S and WS fibers do not melt at 600 °C (Figure 16a,b) and remain in the matrix, they are thought to shorten the expansion caused by temperature effects, leading to better high-temperature performance of these concretes compared to the control mixture. Other researchers have reported similar results [91,92,93,94]. The melting of PP fibers at 160–170 °C, creating extra voids in the matrix (Figure 16c), has improved high-temperature resistance compared to the control mixture through the mechanism of minimizing osmotic pressure due to the temperature effect [95].

PP fiber concretes performed better at 600 °C compared to the other two fiber types in terms of high-temperature resistance. It is known that a high amount of osmotic pressure is generated in the matrix at this temperature. The fact that S and WS fibers remain unmelted prevents the diffusion of vapor pressure, and the mismatch in thermal expansion coefficients between the fibers and the concrete matrix [96] triggers crack formation at the interface regions (Figure 16).

At elevated temperatures, particularly within the range of 600–900 °C, RCC experiences considerable physicochemical alterations that severely compromise its structural integrity [70]. The dissociation of CH, which initiates around 400–500 °C and becomes pronounced beyond 600 °C, results in the release of free water and the generation of CaO, a phenomenon that is both endothermic and structurally destabilizing. Simultaneously, the C-S-H gel, which serves as the primary binding phase in cementitious composites, undergoes gradual depolymerization and densification, yielding diminished cohesive strength and heightened microcracking [70,71,73,84]. These transformations are further intensified by thermally induced mineral phase transitions, such as the conversion of ettringite and monosulfate phases into more stable anhydrous forms, and in certain instances, the crystallization of wollastonite and additional silicate phases [89]. Such modifications not only disrupt the microstructure but also lead to a reduction in strength through increased porosity and interfacial deterioration between aggregate and paste [36,71]. Comparative investigations have revealed that the degree of microstructural damage and residual strength reduction is closely associated with the extent of CH decomposition and C-S-H structural degradation, thereby highlighting the imperative necessity for thermally resilient binders and additives in high-temperature applications of RCC.

**Figure 17:**
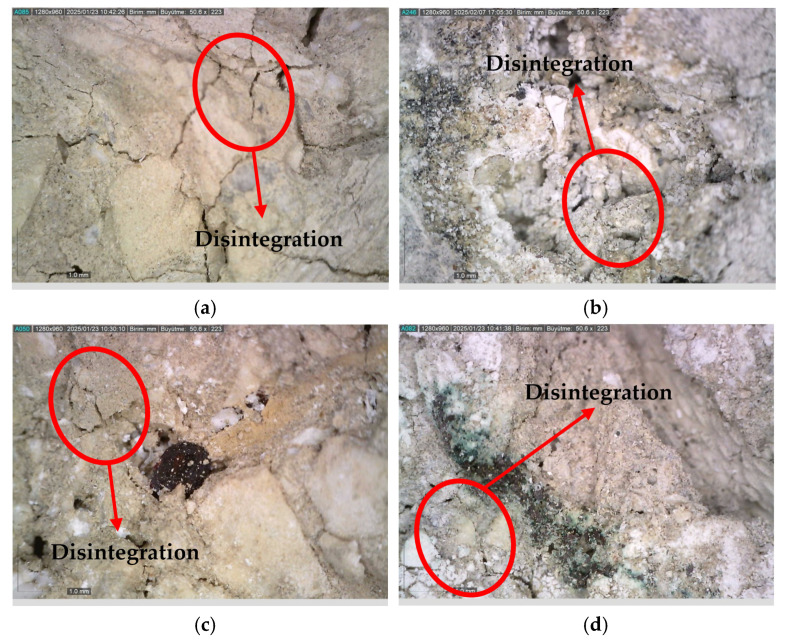
Disintegration of aggregates in RCC exposed to 900 °C: (**a**) control mixture; (**b**) S fiber-reinforced concrete; (**c**) WS fiber-reinforced concrete; (**d**) PP fiber-reinforced concrete.

As the temperature increased from 25 °C to 900 °C, average compressive strength reductions of 91%, 85%, 86%, and 91% were detected for the control, S, WS, and PP fiber concretes, respectively (Figure 13c). During this extreme thermal degradation process, the loss of chemically bound water in the C-S-H gel, the primary binding phase of the concrete, and the breakdown of limestone aggregates [97] have been identified as the primary mechanisms causing strength loss (Figure 17). In particular, the approximately 40% volume expansion caused by the reaction of calcium oxide (CaO) with moisture and CO_2_, forming Ca(OH)_2_ and CaCO_3_ during the cooling phase [36], is the primary reason for the observed structural collapse in the specimens (Figure 6).

The high-temperature performance of S and WS fiber concretes has been found to be superior to that of the control and PP fiber concretes. In fiber-reinforced concrete, although S and WS fibers remained intact at 900 °C, the intensification of thermal mismatches at the fiber–matrix interface [98] and macrocracks on the fiber surfaces (Figure 17) were identified as critical factors accelerating strength loss. These findings suggest that the performance of concrete at ultra-high temperatures is primarily determined by aggregate stability and the durability of the interface region.

#### 3.2.4. Change in Water Absorption Capacity of RCC After Exposure to High Temperatures

**Figure 18:**
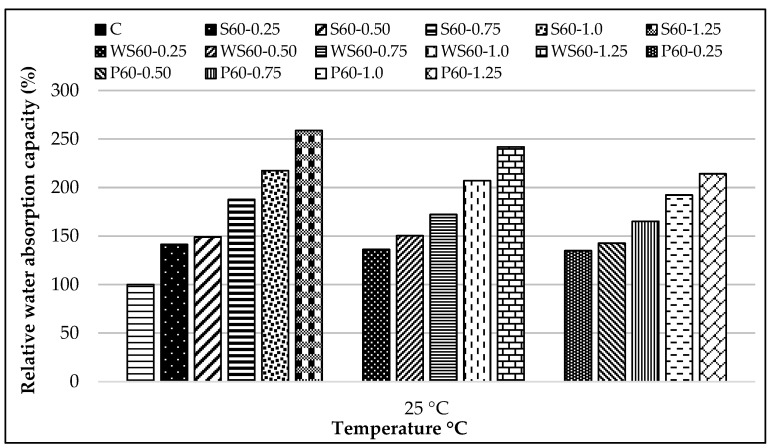
The relative water absorption results at 25 °C of all RCCs compared to the control mixture.

**Figure 19:**
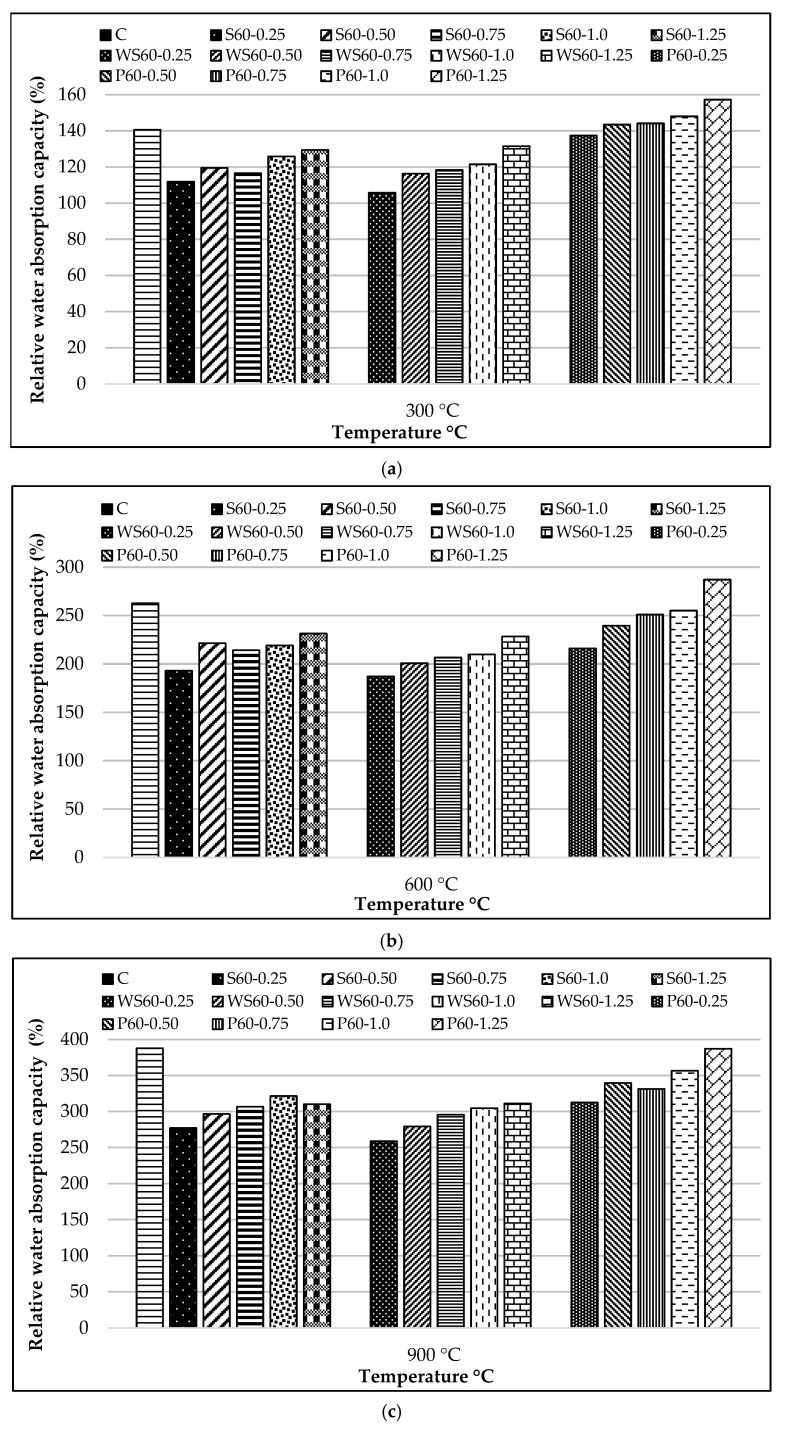
Relative water absorption capacity of samples exposed to different temperatures compared to those at 25 °C: (**a**) 300; (**b**) 600; (**c**) 900 °C.

The relative water absorption capacity results of all RCCs at 25 °C compared to the control mixture are presented in Figure 18, while the relative water absorption capacity of specimens exposed to 300, 600, and 900 °C compared to those at 25 °C is shown in Figure 19a–c.

Regardless of fiber type, a systematic increase in the water absorption capacity of RCC was observed as the fiber dosage increased. S fibers exhibited a 41–158% increase, WS fibers 36–141%, and PP fibers 34–114%. This increase is attributed to the difficulty in compaction due to fiber addition and the increased void content caused by fiber entanglement [79]. The higher water absorption values of S and WS fiber-reinforced RCC are considered an indicator of the difficulty in compaction due to the stiffer structure of these fibers. Furthermore, the smooth surfaces of steel fibers may negatively affect fiber–matrix bonding, increasing void content and contributing to the rise in water absorption capacity. Various researchers have reported similar results [80,99,100,101,102]. It has been observed that WS fiber-reinforced concretes generally exhibit lower water absorption values than steel fiber-reinforced ones. This is thought to be due to the lower number of voids formed by WS fibers during compaction compared to hooked-end steel fibers. On the other hand, concretes containing PP fibers were found to have relatively lower water absorption values. This is believed to be associated with the flexible nature of PP fibers, which lessens void formation [79,103]. These results are consistent with similar studies in the literature, which report a 5–80% increase in water absorption capacity in RCC prepared with different types of fibers [31,104,105].

In specimens exposed to 300 °C, the rate of increase in water absorption capacity varied depending on the fiber type. In S, WS, and PP fiber-reinforced concretes, increases of 11–29%, 5–31%, and 37–57%, respectively, were observed, while the control mixture showed a 40% increase (Figure 19a). At this temperature level, the evaporation of chemically bound water between C-S-H gel layers, leading to shrinkage and layer compaction, is considered the main reason for the increase in water absorption capacity [106]. Especially in the case of PP fibers, the formation of micro-voids due to fiber melting (Figure 15) and the osmotic pressure effect during evaporation explained the marked increase in water absorption.

At 600 °C, the increase in water absorption capacity became more pronounced. Increases of 115–155%, 87–146%, and 111–159% were observed for S, WS, and PP fiber-reinforced concretes, respectively, while the control mixture reached an increase of 181% (Figure 19b). At this stage, the decomposition of Ca(OH)_2_ and thermal expansion of aggregates [107,108] caused significant damage to the concrete’s microstructure. The disintegration of limestone aggregates [97,108] and the osmotic pressure created by water vapor critically increased the void content. The hooked-end structure of steel fibers [79] and compaction difficulty [109] were identified as the main reasons for the differences in water absorption capacity.

At 900 °C, all concretes showed a significant increase in water absorption capacity. S, WS, and PP fiber-reinforced concrete exhibited increases of 177–221%, 158–211%, and 212–287%, respectively, while the control mixture reached a 288% increase (Figure 19c). At this stage, irreversible degradation of the main concrete components and the complete disintegration of aggregates are the main causes of the excessive increase in water absorption. Additionally, the pore networks formed by the complete evaporation of PP fibers and the effect of osmotic pressure are the key factors determining the behavior at this temperature level.

### 3.3. Statistical Evaluation

#### 3.3.1. Taguchi Method

In the experimental study, the measured compressive strength values for all combinations of control factors were analyzed using the Taguchi design method [54].

The obtained S/N ratios and average compressive strength values (means) are presented in Table 9.

The effect of each control factor on the strength performance was analyzed using an “S/N response table”. The S/N response results for strength performance are presented in Table 10.

The results obtained through the Taguchi analysis method highlight the critical parameter combinations for optimizing the mechanical performance of RCC. The analyses show that a volumetric fiber dosage of 1.0% (Factor A, Level 5) maximized mechanical properties by ensuring a homogeneous distribution within the matrix. The identification of room temperature (Factor B, Level 1) as the optimum condition aligns with the disruptive effects of high temperatures on hydration products. The highest performance exhibited by steel fibers (Factor C, Level 2) can be explained by their high elastic moduli and thermal stability properties. This optimum parameter combination demonstrates the ability of the Taguchi method to minimize experimental variations while maximizing performance criteria. This finding provides a reliable guide for RCC design in industrial applications, offering an essential reference for producing high-strength and temperature-resistant concrete.

#### 3.3.2. ANOVA Method

In this study, ANOVA was applied to evaluate the effects of fiber type, dosage, and temperature parameters on the strength properties of RCC. The analysis results, conducted at a 5% significance level and with a 95% confidence interval, are presented in Table 11.

According to the ANOVA results, the statistical significance of the control factors is described as follows: The statistical significance criterion was based on the threshold *p* < 0.05. The analysis findings show that the temperature factor (Factor B) is the most influential parameter on the compressive strength of RCC, with the highest effect at a contribution rate of 75.02%. The fiber dosage (Factor A) was the second most influential parameter with a contribution rate of 17.31%, while the effect of fiber type (Factor C) remained relatively low at 1.16%. The model’s error rate was calculated as 6.51%, supporting the results’ reliability.

## 4. Conclusions

The results obtained in this study are summarized below:

Fiber addition negatively affected the workability of concrete mixtures and manifested through an increase in the Vebe time. This effect became more pronounced as the fiber content increased. Under high-temperature conditions, significant increases in water absorption capacity were recorded in all samples. Especially at the 600 °C temperature level, concretes containing steel, waste steel, and polypropylene fibers showed an average water absorption increase of 131%, 128%, and 187%, respectively, while these increases were found to be even more pronounced at 900 °C.

In terms of mechanical performance, fiber-reinforced concretes exhibited higher strength at 25 °C and 300 °C compared to the control mixture. However, at high temperatures like 600 °C and 900 °C, the strength loss observed in polypropylene fiber concretes was higher than in concretes containing steel and waste steel fibers. In weight loss examinations, at 600 °C, the average weight losses for concretes containing steel, waste steel, and polypropylene fibers were 6.5%, 5.5%, and 7%, respectively, while at 900 °C, these losses ranged between 9% and 16%.

Steel and waste steel fibers helped limit the formation of microcracks in the concrete matrix, thereby preserving mechanical strength. In contrast, the melting of polypropylene fibers at high temperatures led to the formation of voids within the matrix. Overall, it was determined that steel and waste steel fibers exhibited superior performance under high-temperature conditions compared to polypropylene fibers.

One of the most important findings of the study is that waste steel fibers, despite showing similar mechanical performance to traditional steel fibers, can be considered as a sustainable alternative contributing to environmental sustainability.

## Figures and Tables

**Table 1:** The chemical composition, mechanical, and physical properties of the cement [36].

Cement Characteristic	Wt (%)	Physical Property		
SiO_2_	18.81	Specific gravity	3.15	
Al_2_O_3_	5.71	Blaine specific surface (cm^2^/g)	3530	
Fe_2_O_3_	3.09	0.045 mm elekte kalan (%)	7.6	
CaO	62.70			
MgO	1.16	Mechanical property		
SO_3_	2.39	Compressive strength (MPa)	1-day	14.7
Na_2_O + 0.658 K_2_O	0.92		2-day	26.80
Cl^−^	0.01		7-day	49.80
Loss on ignition	3.20		28-day	58.5
Free CaO	1.26			

**Table 2:** Physical properties of aggregate.

**Crushed Lime Stone**	**Size (mm)**	**SSD Bulk** **Specific** **Gravity**	**Loose Bulk** **Density (kg/m^3^)**	**Water Absorption Capacity (%)**
	0–5	2.68	1655	1.64
	5–12	2.68	1441	0.51
	12–22	2.69	1405	0.40

**Table 3:** Properties of the S, PP, and WS fibers used in roller-compacted concretes.

Property	Length (l) (mm)	Diameter (d) (mm)	Slenderness (l/d)	Specific Gravity	Tensile Strength (MPa)	Melting Point (°C)
S fiber	60	1	60	7.8	1000–1400	>1500
PP fiber	60	0.9	67	0.91	450–700	162
WS fiber	60	1	60	7.85	800–1200	>1400

**Table 4:** Some properties of roller-compacted concrete and theoretical mix proportions (kg/m^3^).

Series Designation	Optimum Water Content (%)	Maximum Dry Unit Weight	SSD Aggregate			S Fiber	WS Fiber	PP Fiber	Cement	w/c Ratio
			0–5 (mm)	5–12 (mm)	12–22 (mm)					
C	4.97	2345	1236	412	414	0	0	0	300	0.42
S60-0.25	5.07	2372	1227	409	411	19.50	0	0	300	0.43
S60-0.50	5.23	2356	1223	408	409	39.00	0	0	300	0.43
S60-0.75	5.42	2399	1210	403	405	58.50	0	0	300	0.45
S60-1.0	5.47	2387	1196	399	400	78.00	0	0	300	0.47
S60-1.25	5.51	2394	1192	397	399	97.50	0	0	300	0.47
WS60-0.25	4.53	2359	1227	409	411	0	19.63	0	300	0.43
WS60-0.50	4.65	2367	1223	408	409	0	39.25	0	300	0.43
WS60-0.75	5.03	2365	1210	403	405	0	58.88	0	300	0.45
WS60-1.0	5.36	2406	1196	399	400	0	78.50	0	300	0.47
WS60-1.25	4.96	2403	1192	397	399	0	98.13	0	300	0.47
P60-0.25	4.87	2311	1227	409	411	0	0	2.28	300	0.43
P60-0.50	5.35	2325	1218	406	408	0	0	4.55	300	0.43
P60-0.75	5.46	2330	1204	402	403	0	0	6.83	300	0.46
P60-1.0	5.47	2331	1186	395	397	0	0	9.10	300	0.49
P60-1.25	5.66	2301	1182	394	396	0	0	11.38	300	0.49

**Table 5:** Corrected mix proportions (kg/m^3^) used in the production of 1 m^3^ of RCC.

Series Designation	SSD Aggregate			S Fiber	WS Fiber	PP Fiber	Cement	w/c Ratio
	0–5 (mm)	5–12 (mm)	12–22 (mm)					
C	1307	436	438	0	0	0	300	0.42
S60-0.25	1287	429	431	20.46	0	0	300	0.43
S60-0.50	1300	434	435	41.46	0	0	300	0.43
S60-0.75	1265	421	423	61.15	0	0	300	0.45
S60-1.0	1264	422	423	82.43	0	0	300	0.47
S60-1.25	1254	418	420	102.58	0	0	300	0.47
WS60-0.25	1299	433	435	0	20.77	0	300	0.43
WS60-0.50	1297	433	434	0	41.62	0	300	0.43
WS60-0.75	1280	426	429	0	62.30	0	300	0.45
WS60-1.0	1253	418	419	0	82.23	0	300	0.47
WS60-1.25	1250	416	418	0	102.89	0	300	0.47
P60-0.25	1317	439	441	0	0	2.44	300	0.43
P60-0.50	1289	430	432	0	0	4.81	300	0.43
P60-0.75	1268	423	424	0	0	7.19	300	0.46
P60-1.0	1234	411	413	0	0	9.47	300	0.49
P60-1.25	1234	411	414	0	0	11.88	300	0.49

**Table 6:** RCC parameters and their levels [54].

Parameter	Symbol	Level 1	Level 2	Level 3	Level 4	Level 5	Level 6
Fiber dosage	A	0	0.25	0.5	0.75	1	1.25
Temperature (°C)	B	25	300	600	-	-	-
Fiber type	C	PP	S	WS	-	-	-

**Table 7:** Vebe time of RCC.

Fiber Type	Fiber Dosage (%)					
	0	0.25	0.5	0.75	1	1.25
C	33	-	-	-	-	-
S60	-	41	69	83	92	109
WS60	-	39	67	75	89	103
P60	-	37	53	71	81	94

**Table 8:** The water absorption capacities and compressive strengths of RCC.

Temperature	25 °C		300 °C			600 °C			900 °C		
Series Designation	CS * (MPa)	WAC ** (%)	CS (MPa)	WAC (%)	WL *** (%)	CS (MPa)	WAC (%)	WL (%)	CS (MPa)	WAC (%)	WL (%)
C	59.70	1.55	64.53	2.18	1.73	35.67	4.07	5.19	5.53	6.01	10.86
S60-0.25	63.61	2.19	65.18	2.45	1.89	41.09	4.23	5.40	9.09	6.07	11.52
S60-0.50	62.27	2.31	63.71	2.76	2.19	41.58	5.11	5.62	9.46	6.85	11.11
S60-0.75	65.02	2.91	67.66	3.39	2.50	42.81	6.23	6.32	9.49	8.92	11.13
S60-1.0	66.64	3.37	71.55	4.24	3.11	44.95	7.38	7.07	10.12	10.83	10.86
S60-1.25	56.48	4.01	59.03	5.19	3.36	37.04	9.27	7.89	8.93	12.43	12.21
WS60-0.25	62.23	2.11	62.87	2.23	1.97	43.71	3.95	4.88	8.19	5.46	11.30
WS60-0.50	63.98	2.33	64.22	2.71	2.33	45.87	4.67	5.53	8.02	6.51	11.57
WS60-0.75	61.44	2.67	65.15	3.16	2.66	46.31	5.51	6.40	7.55	7.89	11.57
WS60-1.0	65.81	3.21	68.01	3.90	3.07	48.99	6.73	6.25	9.46	9.78	9.53
WS60-1.25	58.25	3.75	61.47	4.93	2.88	43.26	8.56	6.96	9.51	11.67	12.52
P60-0.25	61.12	2.09	57.94	2.87	2.49	46.55	4.51	6.00	6.16	6.53	14.16
P60-0.50	61.73	2.21	56.21	3.17	2.77	47.84	5.29	6.96	4.82	7.50	12.26
P60-0.75	62.16	2.56	57.83	3.69	2.89	46.78	6.42	6.57	5.89	8.48	13.73
P60-1.0	60.77	2.98	55.14	4.41	3.06	48.51	7.60	7.13	5.78	10.62	14.17
P60-1.25	54.10	3.32	47.32	5.22	3.74	43.47	9.53	7.84	4.69	12.85	14.79

* compressive strength, ** water absorption capacity, *** weight loss.

**Table 9:** Experimental results, S/N ratios, and mean values.

Experiment Number	Control Factors			Compressive Strength (MPa)	S/N Ratio for Compressive Strength	Means for Compressive Strength
	Fiber Dosage	Temperature	Fiber Type			
1	0	25	PP	59.70	35.5195	59.70
2	0	300	S	64.53	36.1952	64.53
3	0	600	WS	35.67	31.0461	35.67
4	0.25	25	PP	61.12	35.7237	61.12
5	0.25	300	S	65.18	36.2823	65.18
6	0.25	600	WS	43.71	32.8116	43.71
7	0.5	25	S	62.27	35.8856	62.27
8	0.5	300	WS	64.22	36.1534	64.22
9	0.5	600	PP	47.84	335958	47.84
10	0.75	25	WS	61.44	35.7690	61.44
11	0.75	300	PP	57.83	35.2431	57.83
12	0.75	600	S	42.81	32.6309	42.81
13	1	25	S	66.64	36.4747	66.64
14	1	300	WS	68.01	36.6515	68.01
15	1	600	PP	48.51	33.7166	48.51
16	1.25	25	WS	58.25	35.3059	58.25
17	1.25	300	PP	4732	33.5009	47.32
18	1.25	600	S	37.04	31.3734	37.04

**Table 10:** Response table for S/N and significance for the compressive strength of RCC.

Response for Signal-to-Noise Ratios				Response for Means			
Level	Fiber Dosage	Temperature	Fiber Type	Level	Fiber Dosage	Temperature	Fiber Type
1	34.25	35.78	34.55	1	53.30	61.57	53.72
2	34.94	35.67	34.81	2	56.67	61.18	56.41
3	35.21	32.53	34.62	3	58.11	42.60	55.22
4	34.55			4	54.03		
5	35.61			5	61.05		
6	33.39			6	47.54		
Delta	2.22	3.25	0.26	Delta	13.52	18.97	2.69
Rank	2	1	3	Rank	2	1	3

**Table 11:** ANOVA results for compressive strength.

Source	Degree of Freedom (DoF)	Sum of Squares (SS)	Mean Square (MS)	F-Value	*p*-Value	Effect Rates (%)
Fiber dosage	5	325.69	65.14	4.26	0.035	17.31%
Temperature	2	1411.08	705.54	46.09	0	75.02%
Fiber type	2	21.83	10.91	0.71	0.519	1.16%
Error	8	122.46	15.31			6.51%
Total	17	1881.05				100.00%

## Data Availability

The original contributions presented in this study are included in the article. Further inquiries can be directed to the corresponding author.
